# Differential expression of mucosal trefoil factors and mucins in pediatric inflammatory bowel diseases

**DOI:** 10.1186/2194-7791-2-S1-A5

**Published:** 2015-07-01

**Authors:** Andreas C Jenke, Veronika Boland, Jan Postberg, Silvia Vogel, Daniel Gödde, Matthias Zilbauer, Robert Heuschkel, Stefan Wirth, Kai O Hensel

**Affiliations:** Department of Pediatrics, HELIOS Medical Center Wuppertal, Faculty of Health, Witten/Herdecke University, Germany; Department of Pathology, HELIOS Medical Center Wuppertal, Faculty of Health, Witten/Herdecke University, Germany; Department of Pediatric Gastroenterology, Hepatology and Nutrition, Addenbrooke's Hospital, University Department of Pediatrics, University of Cambridge, Cambridge, UK

## Aims

In the intestinal mucosa trefoil factors (TFF) and mucins (Muc) - primarily produced by goblet cells - are thought to play a major role in providing barrier function during infection and inflammation. To investigate their role in pediatric Crohn's disease (CD) and ulcerative colitis (UC).

## Methods

We obtained mucosal biopsies of children with CD, UC and healthy controls, analyzed genetic expression using real-time PCR and related the outcomes to clinical data. Subsequently, immunohistochemistry was utilized to verify protein expression in biopsy specimens.

## Results

Levels of TFF2 mRNA were lower in inflamed mucosal samples (terminal ileum (TI) and duodenum) of children with CD, but higher in non-inflamed mucosal samples when compared to healthy controls (p < 0.05). Similarly, TFF2 levels in the TI were significantly lower in inflamed UC tissue. Adjustment for goblet cell density revealed slightly less marked, yet significantly different gene expression in IBD and controls. Furthermore, TI expression of TFF2 and Muc2 was inversely correlated with interleukin-8 expression in CD (p = 0.027).

## Conclusion

Our data demonstrate significant changes in Muc and TFF mRNA expression in pediatric patients with IBD suggesting a role in mucosal healing. Further studies are needed to elucidate a potential use as biomarkers for disease progression.

